# Environmental and Prenatal Exposure to Persistent Organic Pollutants and the Risk of Asthma and Respiratory Outcomes Across the Lifespan: A Review of Emerging Epidemiological Evidence and Mechanisms

**DOI:** 10.7759/cureus.110282

**Published:** 2026-06-05

**Authors:** Eftychios Vittorakis, Evangelos Vittorakis, Serafim G Kastanakis

**Affiliations:** 1 Cardiology, General Hospital of Chania “Saint George”, Chania, GRC; 2 Internal Medicine, General Hospital of Chania “Saint George”, Chania, GRC

**Keywords:** polychlorinated biphenyls (pcbs), poor asthma outcomes, pops, respiratory manifestations, serum biomarkers

## Abstract

Persistent organic pollutants (POPs) are environmentally persistent, bioaccumulative contaminants that remain an important global public health concern despite international restrictions on their production and use. Owing to their lipophilicity, biomagnification within food webs, and ability to cross the placental barrier, these compounds may adversely influence immune maturation and respiratory development during critical periods of susceptibility. This narrative review synthesizes current epidemiological, clinical, and mechanistic evidence evaluating the association between prenatal and lifelong POP exposure and respiratory health outcomes.

A comprehensive literature search was conducted using literature databases to identify relevant studies published between 2000 and 2025. The evidence base included major prospective birth cohorts, such as HELIX, BAMSE, ALSPAC, Generation R, COPSAC, and the Hokkaido Study, in addition to longitudinal, cross-sectional, case-control, and experimental investigations.

Current evidence suggests that prenatal and early-life exposure to POPs, particularly hexachlorobenzene (HCB), dichlorodiphenyldichloroethylene (DDE), and dioxin-like polychlorinated biphenyl congeners, is associated with increased risks of asthma, wheezing, allergic disease, airway obstruction, and recurrent respiratory infections. Mechanistic studies support biological plausibility through immune dysregulation, aryl hydrocarbon receptor activation, oxidative stress, inflammatory signaling, endocrine disruption, and epigenetic modifications. However, findings remain heterogeneous, and causal inference is limited by methodological variability, exposure misclassification, co-exposures, and residual confounding. Further longitudinal studies are required to clarify underlying mechanisms.

## Introduction and background

Persistent organic pollutants (POPs): definition and regulatory context

POPs are a class of environmental contaminants distinguished by their remarkable environmental persistence, pronounced lipophilic bioaccumulative potential, progressive biomagnification throughout trophic food chains, and prolonged biological and environmental half-lives that may extend from several days to multiple decades [1-4]. Whether intentionally produced or generated as unintended industrial byproducts, POPs persist in environmental and biological systems, where they accumulate over time and may contribute to a wide range of adverse health outcomes [1]. The Stockholm Convention on Persistent Organic Pollutants has implemented regulatory measures aimed at banning, restricting, or eliminating the production and use of several highly hazardous POPs, including polychlorinated biphenyls (PCBs), dichlorodiphenyldichloroethane (DDE), polybrominated diphenyl ethers (PBDEs), and hexachlorobenzene (HCB) [3]. Non-compliance with the Stockholm Convention on Persistent Organic Pollutants is not simply due to countries ignoring the treaty. Many countries struggle to fully implement it because of limited technical capacity, weak monitoring systems, and insufficient funding. Nevertheless, despite the implementation of extensive international regulatory restrictions, these compounds continue to persist ubiquitously within environmental matrices and remain an ongoing and evolving threat to human health due to their sustained environmental stability, bioaccumulative properties, and long-term toxicological effects [5].

Types of POPs and their industrial uses

Commonly studied POPs in health research include PCBs, dichlorodiphenyltrichloroethane (DDT) and its metabolite DDE, dioxins and furans (PCDDs/PCDFs), and HCB. Other frequently investigated compounds are PBDEs, per- and polyfluoroalkyl substances (PFAS), organochlorine pesticides (e.g., aldrin, dieldrin, chlordane), and hexabromocyclododecane (HBCD). Among these, dioxins (PCDDs) are considered the most harmful due to their extreme toxicity, persistence, and strong links to cancer, immune dysfunction, and endocrine disruption. Studies mainly focus on four POPs: HCB, DDT and its metabolite, and PCBs, due to their widespread use, persistence, and well-documented toxicological impacts. PCBs are synthetic anthropogenic compounds extensively manufactured for use in a wide range of everyday industrial and commercial applications and, unlike naturally occurring environmental chemicals, are not found naturally in the environment [2]. Different degrees of chlorination among PCB congeners are associated with distinct biological and toxicological effects, while certain congeners exhibit dioxin-like activity, thereby inducing thymic atrophy and profound suppression of immune system function [2]. HCB was historically utilized in a wide variety of agricultural and industrial applications, including as a fungicidal agent for crop seed treatment, in the manufacturing of rubber, aluminum, and industrial dyes, as a pesticide, and as a preservative in wood treatment processes [2-4]. DDT, whose potent insecticidal properties were first recognized in 1939, was subsequently manufactured and extensively utilized on a global scale, whereas DDE and dichlorodiphenyl dichloroethane (DDD) are major metabolic degradation products generated through aerobic and anaerobic biotransformation processes mediated by fungi and bacterial microorganisms [2]. Despite the extensive characterization of these compounds, important gaps remain in understanding how heterogeneous POP mixtures and varying exposure windows influence respiratory outcomes across the lifespan. In particular, existing literature has not consistently integrated prenatal and lifelong exposure data with mechanistic insights, highlighting the need for a more comprehensive synthesis, which the present review aims to address [2-4].

Routes and sources of human exposure

Human exposure to POPs occurs predominantly through dietary intake, particularly via the consumption of contaminated meat, fish, and dairy products; however, additional exposure pathways include ingestion of contaminated materials, inhalational exposure, and dermal absorption through direct environmental contact [5,6]. Researchers have identified POPs at considerable concentrations, particularly within indoor dust environments, owing to their remarkable environmental persistence, resistance to degradation, and prolonged accumulation within enclosed settings [5-8]. Several household and industrial materials have been identified as potential reservoirs or sources of human exposure to POPs, particularly in older products and building materials. These include certain electrical equipment, transformer and capacitor oils, hydraulic fluids, construction materials, sealants, paints, and other materials known to contain or release POPs under specific environmental conditions [8-11]. Electronic waste (e-waste) processing activities conducted in close proximity to residential areas represent a frequent and significant source of environmental contamination and human exposure to hazardous toxicants [7]. In the context of atmospheric pollution, PBDEs and PCBs can adsorb onto fine particulate matter (PM10), enabling their deep penetration into the bronchial tree and lower respiratory tract following inhalation exposure [12-14]. PM10, generated from anthropogenic activities such as fossil fuel combustion, industrial emissions, and long-range transport of Saharan dust, also plays a key role in the environmental redistribution of HCB, DDE, and PCBs within urban ecosystems [2,13-17].

Developmental and early-life exposure

Human exposure to POPs during critical developmental windows is of particular clinical and toxicological concern. Maternal body burdens of these compounds may be transmitted prenatally through placental transfer and postnatally through lactational exposure via breast milk, thereby contributing to early-life exposure during vulnerable stages of development [18-21]. These compounds are characterized by remarkable persistence both within environmental matrices and in human biological systems [21]. Furthermore, the placental barrier provides only partial protection against fetal exposure, permitting the transplacental transfer of compounds such as perfluorooctanoic acid (PFOA) and various PCB congeners [22,23]. Neonates may already carry measurable concentrations of environmental toxicants at birth due to prenatal exposure. Postnatal exposure may further occur through lactational transfer via breast milk and, to a lesser extent, through contaminated infant formula and other dietary sources during early life [24]. Long-term respiratory dysfunctions observed in later stages of development may be partly attributable to prenatal exposure to pollutants, which can cross the placental barrier from the mother’s bioaccumulated body burden to the developing fetus [20]. Overall, early-life exposure to POPs through placental and lactational transfer represents a critical pathway linking maternal body burdens to long-term developmental and respiratory health risks in offspring [18-24].

Respiratory and immune system effects

Accumulating evidence suggests that prenatal and environmental exposure to POPs may significantly influence the development and functional maturation of both the immune and respiratory systems, thereby contributing to the pathogenesis and progression of various disease states [20]. Asthma represents the most prevalent non-communicable disease among the pediatric population and is characterized by airway hyperresponsiveness, chronic airway inflammation, recurrent wheezing episodes, and excessive mucus production [25]. The term asthma encompasses a heterogeneous group of respiratory allergic disorders primarily associated with dysregulation and functional imbalance of T-helper 1 (Th1) and T-helper 2 (Th2) lymphocyte-mediated immune responses [25]. In general, early-life exposure to environmental contaminants is increasingly recognized as a contributing factor in immune and respiratory dysregulation, with a particular relevance to the development and exacerbation of childhood asthma.

Epidemiology and clinical manifestations of asthma

Asthma is one of the most common chronic respiratory diseases worldwide, affecting approximately 8.2% of adults and 9.4% of children in the European Union [9]. Higher prevalence rates in urban populations suggest an important contribution of environmental factors to disease development [9]. Characterized by chronic airway inflammation and hyperresponsiveness, asthma manifests with wheezing, cough, chest tightness, and airflow limitation. Growing evidence indicates that environmental contaminants, including POPs, may contribute to the development and progression of asthma and related respiratory conditions [9].

Limitations of current evidence

Despite growing evidence linking POP exposure to adverse respiratory and allergic outcomes, several limitations hinder definitive conclusions. A major challenge is the heterogeneity of findings across studies, with some investigations reporting significant associations with asthma, wheeze, respiratory infections, or allergic sensitization, while others report weaker or inconsistent relationships. Such variability may reflect differences in exposure levels, pollutant mixtures, timing of exposure, population characteristics, and susceptibility factors. An additional limitation concerns causal inference. Most available evidence is derived from observational studies, which can identify associations but cannot fully establish causality. Although prospective birth cohorts provide stronger temporal evidence, residual confounding and the complexity of real-world environmental exposures remain difficult to eliminate. Individuals are typically exposed to multiple pollutants simultaneously, making it challenging to isolate the contribution of specific POPs to respiratory disease development. Furthermore, while experimental and mechanistic studies support biological plausibility through evidence of immune dysregulation, inflammatory responses, and endocrine-disrupting effects, the precise pathways linking POP exposure to respiratory disease have not yet been fully elucidated. Consequently, although the overall body of evidence suggests a potential role for POPs in respiratory morbidity, further longitudinal and mechanistic research is needed to strengthen causal inference and clarify the underlying biological mechanisms.

Aim of the review

The aim of this narrative review is to critically examine and synthesize the current evidence regarding the relationship between exposure to POPs and respiratory health outcomes throughout the life course, with particular emphasis on vulnerable developmental periods such as prenatal life, infancy, and childhood. Although the adverse health effects of POPs have been extensively investigated in relation to neurodevelopmental, endocrine, and metabolic disorders, their potential contribution to respiratory and allergic diseases remains comparatively fragmented across the literature and has not been comprehensively evaluated within a unified respiratory health framework. This review seeks to address this gap by integrating evidence from epidemiological studies, birth cohorts, population-based investigations, and experimental research examining respiratory, allergic, and immunological outcomes associated with POP exposure. Particular attention is given to asthma, wheezing disorders, respiratory infections, allergic sensitization, and impaired pulmonary function, as well as to the biological processes that may underlie these associations. By bringing together findings that are currently dispersed across environmental health, toxicology, immunology, and respiratory medicine literature, this review provides an updated and focused assessment of the evidence linking POP exposure to respiratory disease susceptibility. Furthermore, this review aims to identify areas of consistency and uncertainty within the existing literature, highlight important knowledge gaps, and outline priorities for future research. In doing so, it contributes to a more integrated understanding of how environmental exposure to persistent pollutants may influence respiratory health and immune function, thereby informing both future scientific investigation and environmental public health strategies.

## Review

Materials and methods

Search Strategy

This study was conducted as a narrative review of the existing literature examining the association between POPs and respiratory health outcomes, including asthma, wheezing, allergic disorders, impaired pulmonary function, and respiratory infections. A comprehensive literature search was performed using PubMed, Scopus, and Web of Science databases, targeting studies published between 2000 and 2025, with particular emphasis on recent epidemiological birth cohort studies and mechanistic investigations. In addition, relevant publications were identified through manual screening of reference lists from included articles and pertinent reviews to ensure comprehensive coverage of the available evidence. As a narrative review, the primary objective of this work was to synthesize and critically appraise emerging evidence, providing an integrative overview of the current state of knowledge rather than conducting a systematic review or meta-analysis.

Search Terms

Primary search terms included “persistent organic pollutants,” “POPs,” “polychlorinated biphenyls (PCB),” “DDE,” “hexachlorobenzene (HCB),” “prenatal exposure,” “environmental exposure,” “asthma,” “wheezing,” “respiratory outcomes,” “allergic rhinitis,” “eczema,” “airway inflammation,” “immune dysregulation,” and “respiratory infections.” Boolean operators, including “AND” and “OR,” were systematically applied to appropriately combine and refine search queries, thereby enhancing specificity and ensuring a focused retrieval of relevant literature.

To enhance transparency and reproducibility of the search strategy, detailed combinations of search terms were systematically constructed using Boolean operators. Specifically, core exposure-related terms (e.g., “persistent organic pollutants,” “POPs,” “PCBs,” “DDE,” “HCB”) were combined with outcome-related terms (e.g., “asthma,” “wheezing,” “respiratory outcomes,” “allergic diseases,” “respiratory infections”) using the operator “AND” to ensure retrieval of studies addressing both exposure and health outcomes. Synonymous and related terms within each category were linked using the operator “OR” to maximize sensitivity of the search. Representative search strings included combinations such as (“persistent organic pollutants” OR “POPs” OR “PCBs” OR “DDE” OR “HCB”) AND (“asthma” OR “wheezing” OR “respiratory outcomes” OR “allergic rhinitis” OR “eczema” OR “respiratory infections”). These structured query formulations were adapted for each database to optimize retrieval while maintaining consistency across platforms.

Study Selection

Studies were selected based on their relevance to respiratory, allergic, and immunological outcomes associated with exposure to POPs across critical life stages, including prenatal development, infancy, childhood, adolescence, and adulthood. The study selection process was conducted as part of a narrative literature review and focused on identifying the most relevant and methodologically robust evidence available regarding the relationship between POP exposure and respiratory health. Literature searches were performed during the previous year using major biomedical and scientific databases, including PubMed, Scopus, Web of Science, and Google Scholar, supplemented by manual screening of reference lists from key articles and review papers. Search terms included combinations of keywords related to POPs, such as PCBs, DDT, DDE, HCB, dioxins, furans, and organochlorine pesticides, together with respiratory and immunological outcomes including asthma, wheeze, respiratory infections, allergy, allergic sensitization, immune dysfunction, and lung function impairment. Eligible studies included prospective birth cohorts, longitudinal investigations, case-control studies, cross-sectional analyses, and population-based epidemiological studies examining associations between POP exposure and respiratory or immunological outcomes [25-28]. Experimental animal studies and mechanistic in vitro investigations were additionally included to provide biological context and to elucidate potential pathways linking POP exposure to disease development. Studies were excluded if they did not evaluate POP exposure, lacked relevant respiratory or immunological outcomes, provided insufficient methodological information, or represented duplicate publications. When multiple publications originated from the same cohort or study population, studies were carefully evaluated to avoid duplication of evidence. Priority was given to publications with the most comprehensive exposure assessment, longest follow-up period, or most relevant respiratory outcomes, while additional reports from the same cohort were retained only when they provided distinct information regarding exposure windows, biological mechanisms, or health endpoints. Given the highly specialized nature of the research question and the relatively limited number of available studies directly addressing POP exposure and respiratory health, the review sought to include all relevant evidence identified through the search strategy rather than applying strict numerical restrictions. Although emphasis was placed on recent literature, earlier studies were retained when they represented landmark investigations, major birth cohorts, unique populations, or foundational mechanistic evidence that continues to inform current understanding of POP-related respiratory toxicity [28-34]. Consequently, the final evidence base included studies from well-characterized mother-child cohorts, long-term prospective investigations, adolescent and adult population studies, systematic reviews, and experimental research examining the immunotoxic effects of POPs.

A comprehensive evaluation was conducted of epidemiological evidence derived from prospective birth cohorts, including studies investigating prenatal and early-life exposure to PCBs, DDE, HCB, dioxins, and related compounds, as well as studies examining asthma, wheeze, allergic sensitization, respiratory tract infections, lung function, and allergy-related outcomes later in life. Experimental and mechanistic studies were incorporated to elucidate potential biological pathways linking POP exposure to respiratory disease. These included investigations of inflammatory signaling cascades, cytokine profile alterations, Th1/Th2 immune imbalance, oxidative stress mechanisms, endocrine-disrupting effects, aryl hydrocarbon receptor (AhR) activation, macrophage dysfunction, and immune dysregulation. Furthermore, selected animal studies were reviewed to complement and contextualize human findings, particularly those assessing airway inflammation, eosinophilic responses, altered immune function, and immunotoxic effects induced by compounds such as PCBs, DDE, HCB, and dioxin-like pollutants. A critical consideration in interpreting the association between POP exposure and respiratory outcomes is the potential influence of confounding factors and methodological limitations across included studies. Socioeconomic status (SES) represents a major confounder, as it is closely linked to both exposure levels, including dietary habits, housing quality, and environmental conditions, and respiratory health outcomes such as asthma prevalence and healthcare access. Additionally, co-exposures to other environmental pollutants, including particulate matter, tobacco smoke, indoor allergens, pesticides, traffic-related air pollution, and industrial contaminants, may act synergistically or independently, complicating the attribution of observed effects specifically to POPs. Many studies differed in exposure assessment methods, biological matrices used for POP quantification, outcome definitions, and approaches for controlling confounding variables, thereby contributing to heterogeneity in findings. Variability in study design also affects the strength of the evidence. Cross-sectional studies are valuable for identifying associations between POP body burdens and respiratory outcomes but are inherently limited in their ability to establish temporal relationships or causality. In contrast, prospective cohort studies, particularly birth cohorts, provide stronger evidence by assessing exposure during critical developmental periods and evaluating subsequent respiratory and immunological outcomes over time. Nevertheless, even cohort studies remain susceptible to exposure misclassification, loss to follow-up, residual confounding, and selection bias. To enhance the reliability of the evidence synthesis, greater emphasis was placed on studies employing biomarker-based exposure measurements, prospective designs, adequate sample sizes, and adjustment for major confounding variables [34-40]. Despite these considerations, substantial heterogeneity remained across studies, necessitating cautious interpretation of findings. These methodological differences underscore the need for additional well-designed longitudinal studies with standardized exposure assessment approaches and rigorous control of potential confounding factors to further clarify the role of POP exposure in the development of respiratory and allergic disease.

Inclusion and Exclusion Criteria

Studies were deemed eligible for inclusion if they were published in the English language and investigated the association between prenatal or environmental exposure to POPs and respiratory, allergic, or immunological outcomes. Investigations encompassing pregnant women, infants, children, adolescents, and adult populations were considered, provided that they reported relevant exposure metrics and clinically pertinent outcome data. Particular emphasis was placed on studies assessing asthma, wheezing phenotypes, airway obstruction, allergic sensitization, eczema, allergic rhinitis, objective measures of lung function, and respiratory infectious outcomes.

Sources and pathways of exposure

Environmental Exposure Patterns and Early Influences

POPs are ubiquitous contaminants that can be detected across a wide range of everyday consumer products and environmental matrices. Experimental animal studies suggest that POPs exert biologically active toxic effects resembling those of pesticide exposure and may be more closely associated with alterations in building materials and indoor environmental composition than with direct enrichment of indoor house dust [25-28].

Established environmental risk factors for asthma development include exposure to house dust mites, bacterial components, and fungal biomarkers; however, the evidence supporting a direct causal role of specific indoor chemical pollutants remains inconsistent and is not universally accepted [28]. In contrast, a growing body of literature indicates that early-life exposure to domestic animals, agricultural or farming environments, passive tobacco smoke, and upbringing in larger family units may confer a protective effect against the development of atopic and allergic diseases, although these associations appear to be significantly modulated by underlying genetic susceptibility [29]. Maternal nutritional patterns have also been implicated in shaping neonatal immunological and respiratory outcomes, with recommendations emphasizing the reduction of exposure to PCBs during pregnancy and early life [30]. However, while multiple environmental factors have been implicated in respiratory disease development, the specific contribution of POPs remains insufficiently disentangled from co-exposures, and evidence regarding their causal role is still evolving and, in some cases, conflicting. Importantly, major exposure pathways including dietary intake, indoor environments, air pollution, occupational settings, and maternal-fetal transfer likely contribute differentially to body burden across life stages, underscoring the need for an integrated, evidence-based synthesis of how these routes influence respiratory risk, which this review seeks to provide [25-30].

Epidemiological evidence

Prenatal Exposure and Childhood Asthma

Early-life respiratory vulnerability is increasingly recognized as the result of complex interactions between environmental toxicants, immune maturation, genetic susceptibility, and developmental airway programming. A combination of environmental exposures and intrinsic biological as well as genetic susceptibility has been implicated in the development of childhood asthma; however, the precise etiological basis of the disease remains incompletely elucidated [27]. Current evidence suggests that pathogenic airway inflammation, coupled with dysregulated repair mechanisms in injured airway tissues of susceptible individuals, may be triggered by immune responses to common environmental exposures, thereby promoting sustained inflammatory activity. Over time, these processes contribute to progressive airway dysfunction and structural remodeling. Importantly, such pathogenic mechanisms occurring during critical periods of lung growth in early life may adversely influence airway development and differentiation, ultimately resulting in persistent alterations in pulmonary architecture and function in later life. In individuals with pre-existing inflammatory priming, ongoing exposure may further perpetuate disease activity and increase the risk of severe exacerbations [27]. Epidemiological data further support the influence of environmental context on disease risk, as children in one study residing in urban areas have been shown to exhibit a 1.78-fold higher likelihood of presenting asthma symptoms after adjustment for age and sex [31]. In addition, evidence from birth cohort studies indicates that future offspring asthma medication use is positively associated with maternal serum concentrations of HCB and dioxin-like polychlorinated biphenyl-118 (PCB-118), highlighting a potential role for prenatal exposure in disease susceptibility [20]. If one assumes that the classical atopic pathway does not fully account for the observed associations between wheeze, respiratory infections, and prenatal exposure to DDE or HCB, an alternative hypothesis may be proposed in which immune responses to pathogens play a more central role. This hypothesis may partly explain the current immunophenotypic classification of asthma into eosinophilic, neutrophilic, and paucigranulocytic subtypes [32,33]. Furthermore, variability across studies regarding adjustment for delivery mode highlights an additional methodological consideration, as some cohorts treat cesarean section merely as a confounding variable, whereas others propose it as a potential mechanistic pathway influencing asthma development. Meta-analytical evidence suggests approximately a 20% increased risk of asthma in children born via cesarean section [34]. These findings support the hypothesis that prenatal and early-life exposure to POPs may contribute to asthma susceptibility through interactions between environmental toxicants, immune maturation, and developmental respiratory vulnerability. These observations underscore the importance of considering both developmental timing and environmental context when evaluating mechanisms underlying respiratory disease susceptibility across early life.

Wheezing and Allergic Outcomes

The reviewed outcomes included allergic rhinitis, wheeze, eczema, pruritic rash (itchy rash), asthma, rhinoconjunctivitis, as well as spirometric indices of pulmonary function, namely forced expiratory volume in one second (FEV1) and forced vital capacity (FVC). Airway obstruction is closely and mechanistically associated with wheezing as a clinical manifestation; however, a comprehensive clinical evaluation, including detailed medical history and physical examination, remains essential, as the presence of wheeze is not pathognomonic for asthma [34-36]. Although the precise mechanisms remain incompletely understood, accumulating evidence indicates that environmental contaminants such as POPs may participate in the development and progression of inflammatory allergic diseases. Allergic rhinitis constitutes one of the most prevalent chronic conditions in the pediatric population. Notably, a combined allergic and non-allergic phenotype is observed in approximately 44-87% of affected individuals. Despite its high prevalence, allergic rhinitis is frequently underdiagnosed or insufficiently treated during childhood, thereby adversely affecting quality of life for both the child and their family and contributing to an additional economic burden on healthcare systems through both direct and indirect costs [37]. Within the context of POP exposure, allergic respiratory outcomes such as wheezing, rhinitis, eczema, and rhinoconjunctivitis may reflect broader pollutant-induced alterations in immune regulation and inflammatory responsiveness. Atopy, from an immunological standpoint, is defined by immunoglobulin E (IgE)-mediated hypersensitivity responses. With regard to atopic conditions, asthma development has been reported in approximately one-third of children with atopic eczema. Furthermore, eczema, asthma, and allergic rhinitis (hay fever) commonly coexist within familial and clinical clusters among affected children, particularly those with atopic eczema [38]. Atopic dermatitis is a chronic inflammatory dermatological disorder that is not contagious and cannot be transmitted between individuals [39]. It has further been suggested that the development of asthma is frequently accompanied by both atopic dermatitis and allergic rhinitis [40]. The pathogenesis of atopic eczema is primarily initiated by genetically determined impairment of epidermal barrier function, with disease onset typically occurring within the first year of life. In most cases, atopic eczema demonstrates a self-limiting course, with resolution commonly observed by 12-16 years of age, although it may cause significant morbidity during early childhood [38]. This interrelationship is commonly conceptualized within the framework of the “atopic march,” whereby the presence of eczema or food allergy in infancy is considered predictive of subsequent development of asthma and allergic rhinitis later in life [38]. Several epidemiological studies have suggested that prenatal or early-life exposure to POPs may contribute to eczema and other atopic manifestations, potentially through immune dysregulation and altered inflammatory signaling pathways. These interconnected clinical manifestations provide an important framework for interpreting how environmental exposures may shape disease trajectories across childhood.

Respiratory Infections

Accumulating evidence suggests that prenatal and early-life exposure to POPs may not only influence allergic and respiratory outcomes, but also alter host immune defenses and susceptibility to infectious diseases during childhood. Environmental pollutant exposure in children has become an increasing public health concern, particularly given that approximately 20% of all childhood deaths in developing countries are attributable to acute respiratory infections [39]. Furthermore, evidence from multiple studies, particularly from experimental laboratory investigations and wildlife studies, indicates that POPs exert immunosuppressive effects, thereby increasing susceptibility to infectious diseases [18,41-48]. These findings emphasize the importance of considering environmental toxicants as potential modifiers of early immune competence and childhood vulnerability to communicable diseases.

Sources of Heterogeneity and Study Reliability in Epidemiological Evidence

The variability in findings across studies investigating the relationship between POP exposure and respiratory outcomes can be attributed to important differences in study design, exposure assessment, and population characteristics. Some studies report positive associations between prenatal or early-life POP exposure and asthma or wheezing, while others find weak or no significant relationships, highlighting inconsistencies in the evidence base. These discrepancies may arise from differences in how exposure is measured (e.g., maternal serum levels versus environmental proxies), the timing of exposure assessment, and the specific POP compounds evaluated. Furthermore, variations in outcome definitions, such as physician-diagnosed asthma versus self-reported symptoms, contribute to reduced comparability between studies. In evaluating the reliability of the evidence, prospective cohort studies, particularly well-characterized birth cohorts, are generally more robust, as they establish temporal relationships between exposure and outcome and allow for better control of confounding variables [40-48]. In contrast, cross-sectional and case-control studies are more susceptible to bias, including recall bias and reverse causation, limiting their ability to support causal inferences. Additionally, studies with larger sample sizes, standardized exposure measurements, and comprehensive adjustment for confounders tend to provide more reliable findings. Therefore, greater weight should be given to longitudinal studies with rigorous methodology when interpreting the overall evidence, while acknowledging that heterogeneity across study designs contributes significantly to the observed inconsistencies. The epidemiological evidence provides moderate support for an association between POP exposure and adverse respiratory outcomes, although the strength and consistency of this association vary according to the outcome examined and the population studied. Across the reviewed literature, the most consistent findings emerge from studies evaluating prenatal and early-life exposures, suggesting that critical developmental windows may represent periods of heightened susceptibility to the respiratory and immunological effects of POPs. Associations have been reported not only for asthma and wheezing, but also for respiratory infections, allergic sensitization, eczema, rhinitis, and impaired pulmonary function, indicating that the potential impact of POP exposure may extend across multiple components of respiratory and immune health. Nevertheless, the evidence is not entirely uniform. Stronger associations are generally observed in studies employing biomarker-based exposure measurements and prospective follow-up, whereas weaker or null findings are more frequently reported in studies with indirect exposure estimates, shorter follow-up periods, or broader outcome definitions. Furthermore, differences in the specific POPs investigated, background exposure levels, pollutant mixtures, genetic susceptibility, and environmental contexts may contribute to the variability observed among cohorts. Importantly, conflicting findings often involve differences in the magnitude of reported associations rather than completely opposing conclusions, with relatively few studies suggesting protective or inverse effects. When considered collectively, the epidemiological data reveal a recurring pattern linking POP exposure to disturbances in respiratory and immune function, particularly following exposure during early developmental stages. The consistency of these observations across geographically diverse populations, together with supporting mechanistic and experimental evidence, strengthens the biological plausibility of the association [6,8,40-46]. Although the available evidence does not yet permit definitive causal conclusions, the overall weight of the epidemiological literature supports the hypothesis that POP exposure represents a potentially important environmental determinant of respiratory disease susceptibility and warrants continued investigation.

Recent advances and cohort evidence

Most Recent Breakthroughs

Much of the current epidemiological evidence is derived from European birth cohorts with systematically documented environmental exposure assessments. Several systematic reviews have examined the association between early-life exposure to POPs and subsequent respiratory health outcomes, allergic manifestations, and immune system development across infancy, childhood, and adolescence, with findings generally indicating limited and heterogeneous evidence for adverse effects [40]. Nevertheless, some studies indicate that POPs, particularly pesticide-related compounds, may represent an underrecognized contributor to asthma risk [40]. A substantial proportion of mechanistic and clinical investigations have focused on inflammatory biomarkers and cytokine profiles, which exhibit a strong association with asthma pathophysiology. In this context, prenatal exposure to POPs and the subsequent development of asthma in early childhood has been increasingly explored through biomarker-based approaches aimed at elucidating potential mechanistic links between exposure and disease onset [41-44]. Furthermore, outcomes involving lower respiratory tract infections and acute otitis media have been reported in Canadian birth cohort studies to demonstrate comparable associations with early-life POP exposure [45,46]. A similar relationship between childhood asthma development and POP contamination in indoor dust has also been identified in case-control studies conducted in highly polluted settings, such as Shanghai, China [6,8]. Prenatal exposure to environmental levels of dioxin-like compounds (DLCs) has additionally been associated with alterations in immune function and an increased susceptibility to infant infections, findings that have been proposed as central to the underlying pathogenic pathways of these pollutants [47]. Moreover, both asthma development and infectious respiratory diseases in neonates have been linked to residential proximity to waste sites containing POPs [48]. Evidence also suggests that deviations in circulating POP concentrations, whether elevated or reduced, may be associated with an increased risk of asthma occurrence, underscoring the potential relevance of internal exposure burden in disease development [49]. In a recent investigation evaluating the association between POPs exposure and respiratory health outcomes, no statistically significant relationships were identified with total or specific IgE concentrations or with reduced pulmonary function parameters; however, prenatal exposure to POPs was positively associated with the presence of airway obstruction, suggesting potential early-life vulnerability to pollutant-related respiratory impairment [50]. More recent evidence includes a cross-sectional study conducted in Greenland and a cohort study from the rural Vhembe district of Limpopo, South Africa; in the Vhembe cohort, indoor residual spraying (IRS) programs implemented for malaria control were associated with elevated maternal peripartum serum concentrations of p,p′-DDT, which in turn were linked to increased allergic risk [51,52]. Notably, an association was also observed between such exposure and wheezing among 3.5-year-old children residing in regions with annual DDT application, underscoring the potential respiratory consequences of environmental insecticide exposure [52]. Furthermore, the impact of maternal blood concentrations of PCBs, HCB, and DDE on subsequent respiratory morbidity in offspring remains insufficiently characterized. Accordingly, recent findings reinforce and expand prior evidence supporting the role of POPs in asthma pathogenesis through multiple biological mechanisms and diverse exposure pathways; for instance, Wu et al. demonstrated that POP exposure contributes to the generation of oxidative stress and airway inflammation, both of which are central processes in asthma pathophysiology [53]. Similarly, Kishi et al. reported that exposure during gestation or early life is associated with an increased risk of asthma development, alongside alterations in immune function and endocrine regulation [54]. With regard to specific compounds, Huang et al. showed that exposure to chlorinated paraffins in fine particulate matter is associated with a higher prevalence of asthma symptoms in children [55]. Moreover, occupational data reported by Wielsøe et al. demonstrated a relationship between elevated POP exposure and impaired lung function, as well as increased allergic manifestations [56]. Expanding the scope to plastic-associated environmental contaminants, Landrigan et al. highlighted that plastic-derived POPs may contribute to increased asthma risk under conditions of high exposure burden [57]. Finally, Margetaki et al. reported that the respiratory and developmental effects of prenatal POP exposure may exhibit sex-specific differences in offspring outcomes [58]. The most recent evidence provides stronger biological plausibility for a causal relationship between POP exposure and asthma by demonstrating consistent associations across multiple exposure pathways and mechanistic domains. The principal strength of these newer studies lies in their integration of longitudinal cohort data, objective biomonitoring measurements, and advanced exposome-oriented approaches, which improve exposure characterization and help explain previously heterogeneous findings in the literature.

Large European Birth Cohorts

Longitudinal birth cohorts such as ALSPAC, Generation R, BAMSE, and others have significantly improved understanding of how early-life environmental exposures influence health. A key initiative, Human Early-Life Exposome (HELIX), integrated six European birth cohorts EDEN (France), Rhea (Greece), INMA (Spain), MoBa (Norway), Born in Bradford (United Kingdom), and Kaunas Cohort (Lithuania) into a harmonized research platform to study the impact of exposures such as air pollution, chemicals, diet, and urban environments on child health and development. By combining existing cohorts, HELIX increased statistical power, enabled cross-country comparisons, and advanced exposome and multi-omics research. This cost-effective collaborative model is supported by the European Commission (FP6, FP7, Horizon 2020, Horizon Europe) and national research agencies [58-62].

Immune dysregulation across epidemiological and experimental studies

Eosinophilic Inflammation and AhR-Mediated Responses: AhR Signaling and Dioxin-like Activity

Increasing attention has been directed toward the molecular and immunological mechanisms through which POPs may contribute to airway inflammation, particularly pathways involving eosinophilic responses and AhR-mediated signaling. Eosinophilic accumulation, activation, and subsequent airway injury are driven by the production of eosinophil-specific chemotactic mediators during the allergic airway inflammatory response. Furthermore, prolonged breastfeeding in the context of prenatal PCB exposure has been correlated with elevated IgE concentrations in offspring at seven years of age, as demonstrated in cohorts from populations with relatively high environmental exposure, such as fishing communities [31]. Emerging evidence suggests that asthma pathogenesis involves AhR-mediated signaling pathways, which may be activated following exposure to dioxins and DLCs, thereby contributing to dysregulated immune responses within the respiratory tract. Dioxin-like PCBs exert their biological effects primarily through binding to the AhR, a ligand-activated transcription factor. Upon activation, AhR modulates gene expression and engages in complex crosstalk with other nuclear transcriptional regulators, thereby generating antagonistic and context-dependent interactions that underscore the mechanistic complexity of this signaling pathway [41,62]. These observations support the concept that pollutant-induced receptor signaling may represent a biologically relevant interface between environmental exposure and persistent inflammatory remodeling within the developing respiratory system.

Asthma Phenotypes and Classification

Asthma is increasingly recognized as a heterogeneous disorder comprising multiple phenotypic and endotypic subgroups that differ in their underlying inflammatory mechanisms, clinical manifestations, and susceptibility to environmental pollutants, including POPs, which may contribute to variations in disease onset, progression, and severity. Current evidence suggests that asthma classification models based on inflammatory phenotypes - namely eosinophilic, neutrophilic, and paucigranulocytic subtypes - demonstrate greater utility in excluding asthma than in accurately predicting its occurrence, as reflected by higher negative predictive values compared with positive predictive values. Consequently, these frameworks appear limited in their ability to identify pediatric populations at elevated risk who may benefit from targeted preventive interventions [63]. One of the most extensively described approaches, as reported by birth cohorts, relies on the characterization of inflammatory asthma phenotypes according to induced sputum cellular profiles. Within this classification system, individuals presenting with a sputum neutrophil proportion exceeding 61% are categorized as having neutrophilic asthma, whereas those with eosinophil counts greater than 1% in the absence of neutrophil predominance are classified as eosinophilic asthma. In contrast, subjects exhibiting both sputum neutrophils below 61% and eosinophils below 1% are designated as having paucigranulocytic asthma [64]. Clinically, neutrophilic asthma endotype has been associated with a later disease onset and a lower prevalence of atopic features, thereby supporting its dissociation from classical IgE-mediated pathways [64]. In Scandinavian population-based birth cohort investigations, elevated maternal serum concentrations of HCB and dioxin-like PCB-118 have been positively associated with subsequent asthma medication use in offspring, further supporting the contribution of environmental exposures to heterogeneous asthma-related phenotypes and disease trajectories [20]. These observations underscore the need for more integrative classification frameworks that incorporate inflammatory profiles alongside early-life environmental exposure patterns to better capture asthma heterogeneity and improve risk stratification in vulnerable pediatric populations.

Childhood and Adult Asthma Immunopathogenesis

Understanding the developmental origins of asthma requires an integrated view of how early immune programming and environmental influences interact to shape disease trajectories across the life course. Within the context of the heterogeneous inflammatory endotypes observed in asthma, the precise contribution of the innate immune system remains incompletely characterized. The immunopathogenic pathways underlying asthma may be initiated in childhood and potentially persist into adulthood, particularly if airway inflammatory mechanisms are comparable between childhood-onset and adult-onset disease phenotypes. This hypothesis is supported by the observation that similar therapeutic strategies are effective across both groups, suggesting shared underlying biological processes [32]. However, existing evidence remains inconclusive, as several studies indicate that such immunological trajectories may be more discernible only beyond the age of six years [20,38,65-67]. Consequently, it is not currently feasible to robustly infer a continuous longitudinal pattern based on cytokine profiles and inflammatory biomarkers across developmental stages. Subsequent asthma medication use in offspring has been positively associated with elevated maternal serum concentrations of HCB and dioxin-like PCB-118 in population-based birth cohort investigations [20]. Also, in other studies, the underlying immunological mechanisms are hypothesized to involve reduced neonatal exposure to maternal vaginal microbiota, leading to altered immune maturation, disruption of the Th1/Th2 balance, and an increased propensity toward atopic sensitization mediated by changes in early-life cytokine responses [35]. Also, chronic inflammatory pulmonary disorders frequently exhibit a mixed Th2/Th1 immune signature, reflecting a more complex and heterogeneous immunopathological milieu [35]. Although HCB does not appear to directly induce antigen-specific T-cell sensitization, experimental murine models suggest that macrophage exposure to HCB-derived metabolites, such as tetrachlorobenzoquinone (TCBQ), may subsequently promote activation of both T and B lymphocyte populations. These findings highlight the complexity of asthma pathogenesis and reinforce the importance of longitudinal, multi-system approaches to better delineate how early-life immune and environmental factors converge to influence disease evolution over time.

Studies revealing underlying mechanisms and biomarker studies

Experimental Evidence of Immunotoxicity in POP Exposure Models

Experimental studies investigating POP exposure models have provided important mechanistic insights into the capacity of these environmental contaminants to disrupt immune homeostasis and induce immunotoxic effects across multiple biological systems. Animal studies have shown that POPs exert biological effects that extend beyond their presence as contaminants in indoor dust, with evidence indicating their functional role as bioactive compounds, including pesticide-like and material-associated toxicants rather than merely passive environmental residues [67-77]. Although the precise mechanistic pathways underlying these effects remain incompletely elucidated, multiple investigations have sought to delineate their immunotoxic potential by assessing the consequences of early-life exposure on key immune cell populations, particularly T- and B-lymphocyte subsets, which serve as broad indicators of immunological integrity and functional competence [41]. In parallel, cytokine profiling has been employed to provide more mechanistic and pathway-specific insights into the immunomodulatory effects of environmental exposures [38,78,79]. Converging evidence from both experimental laboratory models [70] and wildlife studies [71,72] suggests that POP exposure is associated with immunosuppressive effects, frequently manifesting as increased susceptibility to infectious diseases [18,70-72]. Comparative toxicological studies in hepatocytes have revealed that human hepatocytes exhibit a higher susceptibility to genotoxic injury, with increased frequencies of DNA strand breaks and micronucleus formation following HCB exposure. In addition, metabolites of PCB3 have been proposed as potential ultimate mutagenic intermediates, while in vivo and in vitro animal studies consistently indicate that HCB, DDE, and PCBs exert immunosuppressive effects, accompanied by increased DNA damage and micronucleus formation [75]. These findings reinforce the concept that POP-related toxicity involves complex interactions between immune dysregulation, genomic instability, and impaired host defense mechanisms, thereby contributing to broader systemic health consequences following environmental exposure.

Immunosuppression, Oxidative Stress, and Systemic Toxicity

Emerging evidence further suggests that POP-associated toxicity extends beyond direct immunological impairment to include oxidative stress-mediated cellular injury and multisystem inflammatory responses affecting peripheral organs. Additional experimental findings indicate that compounds such as DDT and 7,12-dimethylbenz[a]anthracene (DMBA) may induce cellular and chromosomal damage, potentially mediated through enhanced lipid peroxidation driven by elevated reactive oxygen species generation. In these experimental and epidemiological observations, toxicological effects were not confined to classical immunological targets; rather, the liver and kidneys were also implicated, suggesting the induction of a systemic inflammatory response secondary to HCB exposure, accompanied by the upregulation of genes encoding key inflammatory mediators [18]. These observations support the view that POP exposure may contribute to widespread biological dysfunction through interconnected pathways involving redox imbalance, inflammatory signaling, and tissue-specific toxic responses.

Molecular and Signaling Pathways of Immune Disruption

At the molecular level, increasing evidence indicates that POPs interfere with multiple intracellular signaling pathways and immune regulatory mechanisms that are essential for maintaining coordinated inflammatory and antigen-responsive functions. PCB exposure has been demonstrated to modulate critical inflammatory signaling cascades, including inhibition of downstream nuclear factor-kappa B (NF-κB) activation in J774A macrophage cell lines, as well as significant interactions with lipopolysaccharide (LPS)-induced toll-like receptor 4 (TLR4) signaling and CD14 upregulation [18,80-82]. Independent of synergistic pollutant interactions, HCB has also been shown to directly activate macrophages, leading to upregulated transcription of inflammatory genes, as demonstrated by transcriptomic profiling [18]. Mechanistically, impaired antigen presentation has been attributed, in part, to PCB-induced reductions in macrophage endocytic capacity, thereby compromising efficient immune surveillance [82]. Additional experimental evidence supports the capacity of PCBs, particularly PCB3, to function as a potential ultimate mutagen [75]. These mechanistic observations highlight the multifaceted nature of POP-induced immunotoxicity and suggest that persistent disruption of cellular signaling networks may contribute to long-term immune dysfunction and heightened susceptibility to inflammatory disease processes.

Epidemiological Correlates and Clinical Translation

Translational and epidemiological investigations have further expanded the understanding of POP-associated immunotoxicity by linking experimentally observed immune alterations with clinically relevant respiratory and immunological phenotypes in human populations. Several lines of evidence further suggest that immunotoxic and respiratory associations may occur independently of IgE-mediated pathways, as supported by observed relationships between DDE exposure and wheezing in non-atopic pediatric populations [78-82]. Moreover, at the immunological level, humoral immune responses, cutaneous lesions, pulmonary eosinophilia, and increased auricular lymph node (ALN) cellularity have been shown to be induced by HCB in a T cell-dependent manner [18]. Exposure to PCB-118 has been associated with an increased polarization of CD4+ T lymphocytes toward a Th2-skewed immunophenotype, a pattern that has been linked to allergic disease outcomes [45,83,84]. These findings underscore the heterogeneous and multifactorial nature of POP-associated immune dysregulation and support the potential contribution of early-life environmental exposures to long-term respiratory and immunological vulnerability.

Oxidative Stress and Inflammation

Biomarker studies have further demonstrated that interleukin-8 (IL-8) mRNA expression in asthmatic children has been shown to correlate with serum concentrations of specific PCB congeners, including PCB #163 + 164, #170, #177, #178, and #180 + 193 [44]. In addition, evidence suggests that certain molecular targets may be affected through interference with cyclooxygenase-2 (COX-2)-mediated pathways, further implicating these environmental contaminants in the modulation of inflammatory signaling networks [44]. These mechanistic insights support the relevance of pollutant-driven redox imbalance as a potential contributor to altered immune regulation in asthma, warranting further investigation into targeted interventions that address upstream environmental triggers.

Endocrine Disruption

POPs have increasingly been recognized for their capacity to interfere with hormonal regulation, positioning endocrine disruption as a key mechanistic pathway linking environmental exposure to systemic health effects. From an endocrine perspective, estrogenic PCB congeners may exert their effects via competitive binding to estrogen receptors and interference with key enzymes involved in estrogen signaling, whereas anti-estrogenic congeners may suppress endogenous estrogenic activity [81]. Adverse health outcomes associated with background-level exposure to POPs remain a significant public health concern, largely due to their environmental persistence and capacity for bioaccumulation even at low-dose exposure levels [81]. These observations emphasize the need for continued mechanistic and epidemiological research to clarify the long-term clinical implications of low-dose endocrine interference and its contribution to environmentally driven disease burden.

Epigenetic and Developmental Effects

Early developmental exposures, particularly during gestation, are increasingly recognized as critical determinants of long-term immune programming and disease susceptibility through epigenetic and immunomodulatory mechanisms. Maternal dietary exposure has also been increasingly recognized as a critical determinant of infant immunological and respiratory health, with emerging evidence supporting the need to identify and implement strategies aimed at reducing exposure to PCBs during the prenatal and early-life periods, particularly through dietary pathways in newborns and mothers [80]. Furthermore, exposure to POPs has been proposed to skew immune responses toward IL-4- and IL-13-dominant pathways, thereby potentially contributing to in utero immune priming for allergic disease phenotypes. Accumulating evidence suggests that early-life exposure to organic pollutants may be associated with previously described sex-specific effects in asthma susceptibility, a hypothesis increasingly supported by recent studies, underscoring the growing scientific interest in this field [53,55-58]. These observations highlight the potential importance of epigenetic and immunological mechanisms in mediating the respiratory effects of early-life POP exposure; however, the clinical and translational significance of many proposed biomarkers remains uncertain. Although alterations in cytokine profiles, immune-cell differentiation, inflammatory signaling pathways, and epigenetic regulation have been consistently reported in experimental and mechanistic studies, the reproducibility of these findings across different populations, exposure scenarios, and study designs remains incompletely established. Furthermore, many of the identified biomarkers have not yet demonstrated sufficient specificity or predictive value to serve as reliable indicators of future respiratory disease risk at the individual level. A further challenge lies in bridging the gap between mechanistic observations and clinical outcomes. While experimental studies provide compelling evidence that POP exposure can influence immune maturation, inflammatory responses, and developmental programming, translating these molecular alterations into measurable respiratory outcomes such as asthma, wheezing, allergic disease, or impaired lung function remains complex. Not all biological changes necessarily result in clinically significant disease, and the magnitude of their contribution relative to other genetic, environmental, and developmental determinants remains difficult to quantify. Consequently, caution is warranted when interpreting individual biomarkers as direct surrogates of disease susceptibility. Nevertheless, the convergence of mechanistic findings with epidemiological observations strengthens the biological plausibility of a causal relationship between early-life POP exposure and adverse respiratory outcomes [80]. In particular, evidence of immune dysregulation, altered cytokine signaling, and epigenetically mediated developmental programming provides a coherent framework through which the associations observed in birth cohorts and population-based studies may be understood [80-93]. Future research should therefore prioritize the validation of candidate biomarkers in large, well-characterized longitudinal cohorts, evaluate their reproducibility across populations, and determine their capacity to predict clinically relevant respiratory outcomes. Such efforts would not only enhance the translational value of current mechanistic evidence but also contribute to a more comprehensive understanding of how environmental exposures become biologically embedded and influence respiratory health across the life course.

Current challenges and limitations in the literature

Despite the expanding body of evidence linking POPs to asthma, allergic disease, and other respiratory outcomes, several fundamental challenges continue to limit the ability to draw definitive conclusions or achieve full evidence integration. A key unresolved issue is the lack of harmonization in exposure-response frameworks, whereby differences in pollutant mixtures, exposure timing, and biomarker interpretation prevent the establishment of comparable exposure metrics across studies [49-52]. This limits the ability to define consistent dose-response relationships or identify threshold effects across populations. A further major challenge lies in the difficulty of disentangling POP-specific effects from the broader environmental exposome. In real-world settings, exposures occur as complex, dynamic mixtures that vary across geography, time, and socioeconomic context, making it difficult to isolate independent contributions of individual compounds. This complexity is compounded by developmental susceptibility, where effects may depend not only on exposure level but also on timing relative to immune and respiratory system maturation, which is not consistently captured across studies [47,49-52].

Another important limitation is the incomplete translation between epidemiological associations and mechanistic resolution. While experimental studies suggest plausible biological pathways involving immune modulation and inflammatory signaling, these mechanisms are not yet sufficiently integrated with population-level findings to support a unified causal framework. As a result, inconsistencies across studies are not solely methodological in nature but also reflect gaps in mechanistic understanding that hinder causal interpretation. Finally, the field is limited by the absence of integrative analytical approaches capable of synthesizing heterogeneous evidence across study designs, populations, and outcome domains. Current literature remains largely fragmented, with findings distributed across epidemiology, toxicology, and clinical research without a fully coherent framework for integration [49-58]. Addressing these challenges will require approaches that move beyond single-chemical and single-outcome paradigms toward more systems-based and longitudinal perspectives capable of capturing the complexity of POP-related respiratory risk.

## Conclusions

POPs, including PCBs, DDE, HCB, and related compounds, remain important environmental contaminants despite international restrictions on their production and use. Due to their persistence, bioaccumulation, and widespread environmental distribution, human exposure continues to occur through food consumption, indoor dust, air pollution, household materials, and occupational or environmental sources. Across the literature, the most consistent findings relate to exposure during critical developmental periods, particularly pregnancy and early childhood, when the immune and respiratory systems are undergoing maturation. Although several biological mechanisms have been proposed, the available evidence remains insufficient to establish definitive causal pathways linking POP exposure to specific respiratory outcomes.

Interpretation of current findings should be cautious. The literature is characterized by substantial heterogeneity in exposure assessment methods, outcome definitions, study populations, pollutant mixtures, and control of potential confounding factors. While many studies report positive associations, results are not uniformly consistent, and important uncertainties remain regarding the magnitude, timing, and long-term clinical significance of these effects. Future research should prioritize harmonized exposure assessment, standardized respiratory outcome measures, long-term longitudinal follow-up, and integration of biomarker, exposome, and multi-omics approaches to strengthen causal inference and identify susceptible populations. A clearer understanding of the relationship between POP exposure and respiratory health may help inform future risk assessment, environmental policy, and targeted strategies to reduce potentially harmful exposures during vulnerable developmental periods.
